# National Scale-up of Zinc Promotion in Nepal: Results from a Post-project Population-based Survey

**DOI:** 10.3329/jhpn.v29i3.7868

**Published:** 2011-06

**Authors:** Wenjuan Wang, Vicki M. MacDonald, Mahesh Paudel, Kathryn K. Banke

**Affiliations:** ^1^ICF MACRO, 11785 Beltsville Drive, Calverton, MD 20705, USA; ^2^Abt Associates Inc., 4550 N Montgomery Ave., Suite 800N, Bethesda, MD 20814, USA; ^3^Population Services International, Kathmandu, Nepal

**Keywords:** Child health, Diarrhoea, Diarrhoea, Infantile, Knowledge, attitudes, and practices, Oral rehydration solutions, Oral rehydration therapy, Zinc, Nepal

## Abstract

The World Health Organization and the United Nations Children's Fund recommend using a new oral rehydration solution (ORS) plus zinc supplementation for 10-14 days for the treatment of diarrhoea in children aged less than five years. The Social Marketing Plus for Diarrhoeal Disease Control: Point of Use Water Disinfection and Zinc Treatment (POUZN) project in Nepal was one of the first zinc-promotion projects to move beyond pilot efforts into a scaled-up programme with national-level reach. This study used data from a survey conducted in 26 districts in Nepal in 2008 to examine zinc-use behaviour, knowledge, and beliefs of caregivers of children aged less than six years, other diarrhoea-treatment practices, and recollection of project communication messages. The results of the survey indicated that, by six months following the onset of a zinc-promotion campaign, the majority (67.5%) of children (n=289), aged less than six years, with diarrhoea were treated with ORS, and 15.4% were treated with zinc. Over half (53.1%) of all caregivers (n=3,550) interviewed had heard about zinc products; most (97.1%) of those who had heard of zinc knew that zinc should be used for the treatment of diarrhoea. Zinc-related knowledge and behaviours were positively associated with recall of communication messages. Children whose caregivers recalled the mass-media message that zinc should be used for 10 days [odds ratio (OR)=2.02, 95% confidence interval (CI) 1.85-2.19] and whose caregivers perceived that zinc is easy to obtain (OR=1.76, 95% CI 1.49-2.09) were more likely to be treated with zinc for 10 days, along with ORS. The findings demonstrated that mass media play an important role in increasing caregivers’ knowledge about zinc and encouraging trial and correct use. Future efforts should also focus on understanding the factors that motivate providers to continue recommending antibiotics and antidiarrhoeals instead of zinc. These findings are being used for informing the design and implementation of zinc programmes in other developing countries with a high prevalence of diarrhoea.

## INTRODUCTION

Diarrhoea is the second leading cause of mortali-ty of children aged less than five years (under-five mortality) globally, resulting in 1.5 million child deaths each year (1). Since the 1980s, the World Health Organization (WHO) and the global health community have been promoting oral rehydration therapy (ORT) for the treatment of diarrhoea. In response to several studies that demonstrated the effectiveness of ORT, along with zinc supplementation, during bouts of acute and persistent diarrhoea, to reduce severity, duration, and recurrent episodes (2-5), the WHO/United Nations Children's Fund (UNICEF) in 2004 revised their guidelines to recommend the use of a new oral rehydration solution (ORS) with reduced levels of glucose and salt, along with zinc supplementation for 10-14 days, for the treatment of diarrhoea in children aged less than five years (under-five children) (6). Limited published data exist to evaluate the effectiveness of these new guidelines in pilot zinc-promotion programmes (7-9), and less has been published to describe the results of implementing zinc programmes on a larger-scale, except for a programme in Bangladesh (10) and the programme in Nepal that is discussed in this paper.

**Fig. 1.**
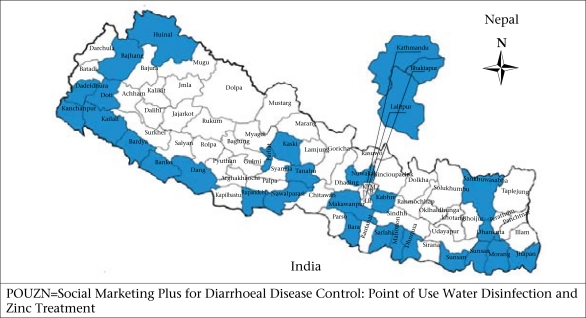
POUZN focus districts (n=30)

The Ministry of Health and Population (MoHP), through its Child Health Division, of the Government of Nepal, was one of the first health ministries to create a Zinc Task Force and prepare stakeholders for the introduction of zinc in line with the new recommendations of WHO/UNICEF. Beginning in 2005, the United States Agency for International Development (USAID) funded the Nepal Family Health Project (NFHP) to provide commodities, training, and technical assistance to strengthen the skills of public-sector healthcare providers and the Social Marketing Plus for Diarrhoeal Disease Control: Point of Use Water Disinfection and Zinc Treatment (POUZN) project to introduce paediatric zinc through private-sector channels. The public-sector pilot zinc programmes began in 2005 in five districts (Kailili, Parbat, Humla, Udayapur, and Bara), and the POUZN pilot began in 2007 in three districts (Kathmandu, Lalitpur, amd Bhaktapur). In 2008, the POUZN project was scaled up to 30 of the 75 districts in the country that were focus districts for community-Integrated Management of Childhood Illness (c-IMCI), representing approximately 50% of the population (Fig. 1).

The objectives of this study were to examine zinc-use behaviour, zinc-related knowledge and beliefs, other diarrhoea-treatment practices, and recollection of project communication messages and to identify the predictors of use of zinc at the end of POUZN project activities in the target districts in Nepal. The POUZN project conducted a household survey at the end of the project to examine these factors both to inform future activities in Nepal and to strengthen the POUZN zinc programmes in other countries.

## MATERIALS AND METHODS

### Programme design

At the beginning of the programme inception, zinc products for the treatment of paediatric diarrhoea were not available in either the public or the private sector. Both UNICEF and POUZN project procured for the MoHP a supply of 20-mg dispersible zinc sulphate tablets (Nutriset/Rodael, France) to cover the initial needs of the public sector; UNICEF and the NFHP ensured distribution to public-sector facilities and trained public-sector healthcare providers at the district and clinic levels in all the 30 focus districts under c-IMCI. Using the manufacturer's model of social marketing (11), the POUZN project encouraged local firms to manufacture dispersible zinc tablets, and by August 2007, three firms had produced, registered, and begun distributing their 10 and 20-mg sulphate-based zinc tablets through their own distribution systems to pharmacies in the 30 project districts and beyond.

Using the training materials developed by the MoHP, the POUZN project trained 5,800 licensed private care providers (98% of whom were chemists) in all the 30 districts and 2,243 public-sector care providers and volunteers in the three Kathmandu Valley districts.

Based on the Health Belief Model (12), the POUZN project developed and aired a national mass-media campaign focused on television and radio (media channels frequently used by Nepali consumers) to promote the use of zinc and ORS. The campaign was launched in April 2008 and ran throughout the diarrhoea season (April-August 2008). In April 2007, a smaller-scale radio campaign was implemented in the initial three POUZN pilot districtsof Kathmandu, Lalitpur, and Bhaktapur. The campaign aimed to ensure that all caregivers of children aged less than six years (i.e. 71 months and below—the target group for MoHP efforts) understood that zinc is an appropriate treatment for diarrhoea, knew that zinc tablets were available from public and private clinics/chemist-shops, and knew that zinc should be used for at least 10 days, along with ORS, to maximize its effectiveness. All media messages linked zinc to continued use of ORS, which had been heavily promoted by the MoHP for several years for the treatment of childhood diarrhoeas. Two national and 19 regional radio stations broadcast radio-spots 16 times daily, and four television stations with national range broadcast a television commercial nine times daily during the diarrhoea season. There were no other parallel programmes or campaigns at the time (or previously), and the media campaign promoted ORS and zinc to potential users of both public and private health services.

### Survey design

#### Survey population and sample

The POUZN project conducted a household survey in August 2008 (end of the diarrhoea season). The survey was designed to cover all the 30 POUZN project districts. However, since four districts (Bajhang, Dadeldhura, Doti, and Humla) were not accessible due to flooding and landslides during the survey period, these were not included.

The sample-size was calculated using a type 1 error rate of 5% and 80% power to detect a 10% increase in use of zinc for the treatment of paediatric diarrhoea, assuming 50% use of zinc at baseline to maximize the sample-size. Based on the assumed paediatric diarrhoea prevalence of 12% (13) and using a designeffect of 2, the required sample-size was calculated as 3,550 households.

#### Sampling method

**Fig. 2.**
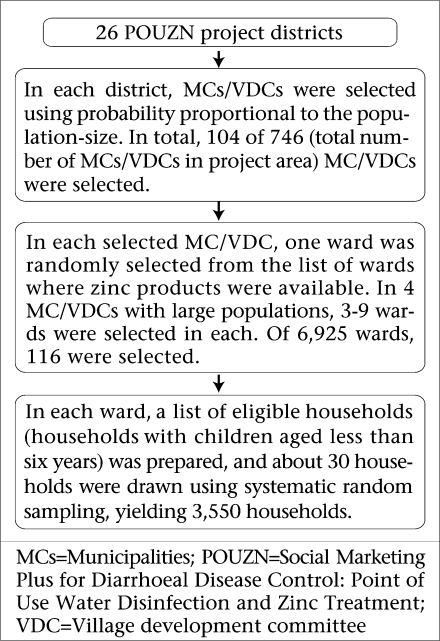
Sampling method of the survey

A multi-stage sampling approach was used for sampling households (Fig. 2). Each district in Nepal is divided into village development committees (VDCs) or municipalities (MCs). Most VDCs are divided into nine wards, depending on the size of the population within a VDC. We treated each of the 26 districts as a sampling stratum. The VDC/MC was the primary sampling unit, the ward was the secondary sampling unit, and the household was the ultimate sampling unit. The desired number of VDCs/MCs was selected using probability proportional to population-size in each district. In each selected VDC/MC, a list of wards in which zinc products were available was prepared. In each of four MCs with large populations—Khandbari MC in Sankhuwasaha district, Lalitpur UMNP (sub-metropolitan city) in Lalitpur district, Kathmandu MNP (metropolitan city) in Kathmandu district, and Pokhara UMNP in Kaski district—3-9 wards were randomly selected. One ward was randomly selected from each of the remaining VDCs/MCs. In each selected ward, 30 eligible households (households with children aged less than six years) were selected using systematic random sampling. In eight wards, one or two additional households were interviewed. Due to refusal or absence of caregivers from home, interviews could not be conducted in 9% of the households, and alternates were selected and interviewed to achieve the desired sample-size. In each selected household, the caregiver of the child(ren) aged less than six years who could best answer the questions about those children who had diarrhoea within the past two weeks was interviewed.

The survey collected data for the youngest and next to the youngest child (if there was one) in each household using an instrument administered in Nepalese. The survey instrument collected information on the sociodemographic characteristics of households and caregivers, diarrhoeal history of children aged less than six years, treatment of diarrhoea with zinc and other therapies, knowledge and beliefs about diarrhoea and different treatments, and recall of programme communication messages.

### Analysis of data

Data were analyzed using the Stata software (version 10.0) (StataCorp, College Station, TX). Des-criptive analysis was used for describing the variables of interest. Bivariate analyses were conducted to investigate the unadjusted association between the variables (i.e. not controlling for other variables that may confound the association). Multiple logistic regressions were employed to examine the adjusted association between use of zinc and potential predictors, controlling for other confounders. We accounted for the complex survey design to estimate efficient coefficients. Sampling weights were computed to reflect the probability of selection into the sample and adjusted for non-response to allow generalizations to the population from which the sample was drawn. The clustering effect at the VDC/MC level was taken into account. There were no differences in our outcome of interest (use of zinc for diarrhoea) in urban and rural populations, and analyses, therefore, combined both the groups.

We developed an asset wealth index using principal component analysis of a set of household characteristics and ownership of durable goods or other possessions (14,15). The wealth index has been shown to be a more reliable socioeconomic proxy than income variables and other quantitative socioeconomic measures in developing countries (16). We divided the population into quintiles based on their wealth index, with the lowest quintile representing the poorest 20% and the highest quintile representing the wealthiest 20% of the households.

In multivariate analysis, we first tested bivariate associations (the association of each predictor with each dependent variable). The variables examined included demographic characteristics of caregivers, household characteristics, knowledge of and attitudes towards zinc products, and recall of communication messages. Variables showing a significant (p<0.05) association with the outcomes were included in the final multivariate regression models.

### Ethical issues

The local Institutional Review Board (IRB) in Nepal did not require review of the study because it is considered exempt research by the Abt Associates Inc. IRB according to USAID guidance. Oral consent was obtained from all the respondents before interview.

## RESULTS

### Background characteristics of study sample

Of the 3,550 households surveyed, 44% were from urban areas and 56% from rural areas. The large majority (72%) of the caregivers were aged 20-30 years, and all were virtually female and Hindu (Table 1). Approximately half of the respondents had five or fewer years of schooling.

**Table 1.** Percentage distribution of respondents (n=3,550) by demographic cha-racteristics and mass-media exposure

Caregivers of children agedless than six years	%
Age (years)	
15-19	4.5
20-24	33.8
25-29	37.9
30-34	14.4
35 and above	9.4
Gender	
Female	99.8
Male	0.2
Education	
No education	35.7
Primary (grade 1-5)*	16.9
Secondary (grade 6-12)†	43.0
Higher‡	4.4
Residence	
Urban	43.8
Rural	56.2
Religion	
Hindu	87.5
Buddhist	6.2
Muslim	3.9
Christian	1.1
Others	1.3
Exposure to mass media	
Frequency of listening to radio	
Daily	43.5
At least once a week but less than daily	15.6
Less than once a week	10.9
Never	30.1
Frequency of watching television	
Daily	63.2
At least once a week but less than daily	9.2
Less than once a week	6.0
Never	21.6
Frequency of reading newspapers or magazines	
Daily	16.9
At least once a week but less than daily	22.7
Less than once a week	21.0
Never	39.5
Main source of drinking-water	
Improved source¶	95.0
Unimproved source	5.0
Primary sanitation facility	
Improved, not shared facility	43.3
Unimproved facility	56.7

*Includes those who completed 1-5 years of schooling;

†Includes those who completed 6-12 years of schooling;

‡Includes those who attended university;

¶Improved water sources refer to piped water, water from protected wells, and water from protected springs

The majority (59%) of the respondents listened to radio at least once a week, with 44% listening to radio daily. More than two-thirds (72%) of the res-pondents watched television at least once a week, and 63% watched television every day. The urban respondents (n=1,555) were somewhat more likely than the rural respondents (n=1,995) to watch tele-vision daily or at least once a week while the rural respondents were more likely to listen to radio daily or at least once a week (data not shown).

The survey areas had better access to improved drinking-water and sanitation facilities than the national average. Most (95%) households obtained drinking-water from an improved source and more than 40% of the families had an improved sanitation facility.

### Use of zinc for treatment of diarrhoea

Overall, we collected data on 4,211 children (3,550 youngest children and 661 next youngest children). Their average age was 31 months, with 22% aged one year or younger and 5% aged 60-71 months. Half of the children were girls, and half were boys. Just over 5% of the children had diarrhoea in the two weeks before the household survey.

The caregivers of children who had diarrhoea in the two weeks preceding the survey were asked about specific treatments used for treating this episode of diarrhoea. Our survey found that 15% of the children with diarrhoea in the last two weeks were treated with zinc in the 26 POUZN project districts (Table 2). Of these users of zinc, 80% received advice from a healthcare provider to use zinc to treat diarrhoea.

**Table 2.** Percentage distribution of treatments for children, aged less than six years, with diarrhoea

Treatment pattern	Children with diarrhoea in the last 2 weeks (n=289)* (%)	Children treated with zinc (n=46) (%)
Treated with zinc	15.4	
Treated with zinc plus ORS	12.1	78.6
Given zinc for 10 days or more†	10.1	64.9
Treated with zinc for 10 days or more plus ORS	8.3	53.9
ORS‡	67.5	
Recommended home-fluids	56.5	
Antibiotics	11.8	
Antidiarrhoeals	17.7	
Unknown pills or syrups	60.0	
Home-remedy	17.6	
No treatment	6.8	

*Total may exceed 100% because the respondents were allowed to state multiple treatments;

†Denominator includes two children who took zinc for fewer than 10 days but still had diarrhoea on the day of the survey and, thus, could potentially complete a full 10-day course;

‡Includes those respondents who used both ORS and zinc.

ORS=Oral rehydration solution

Just 8.3% of the children with diarrhoea were treated with zinc for 10 days and ORS as recommended. However, compliance with correct zinc administration was generally good among the users of zinc. Adherence to the 10-day regimen was high: 65% of 46 children who received zinc did so for at least 10 days (Denominator includes two children who took zinc for fewer than 10 days but still had diarrhoea at the time of the survey and, thus, could potentially take the full 10-day course), and 79% of the children who received zinc were also treated with ORS. Just over half (54%) of the users of zinc were given both zinc for 10 days and ORS as recommended. Lack of knowledge of the importance of using zinc together with ORS was the main reason reported by those caregivers who used zinc without ORS (75%).

The users of zinc obtained the zinc tablets primarily from private pharmacies (32%), health posts (29%), private clinics (26%), hospitals (18%), and female community health volunteers (FCHVs) (15%).

### Other treatments for diarrhoea

Children with diarrhoea were given various treatments other than zinc. The most commonly-reported treatment given to children with diarrhoea was ORS (68%). More than half (57%) of the children were given recommended home-fluids, such as rice-water, sugar-salt-solution, or lentil soup. Only 6.8% of the children with diarrhoea did not receive any treatment; “the child was not very sick” was cited by 73% of the respondents as the reason for not providing treatment.

Sixty percent of the children were given an unknown pill or syrup. The study found that an antibiotic was given to 12% of the children with diarrhoea, only one-fifth of whom reported blood in the stool. Antidiarrhoeals should only be given to adults or children aged over 10 years. However, 18% of the children in this survey were given antidiarrhoeals. In addition, 18% were treated with other home-remedies.

### Knowledge and beliefs about zinc

The study found that 53% of the respondents had heard about zinc products. Of them, 38% were aware that zinc should be given to children with acute diarrhoea, and 80% knew that zinc should be given to children with persistent diarrhoea (Table 3). Fifty-three percent knew that zinc should be given for at least 10 days to a child with diarrhoea. Knowledge was linked to adherence: 71% of the res-pondents who were aware of the 10-day regimen actually followed it (data not shown).

**Table 3.** Knowledge and beliefs about zinc among caregivers of children aged less than six years

Knowledge and belief	% of caregivers who had heard aboutzinc (n=1,772)	% of caregivers who treated children with zinc (n=37*)
Knowledge about zinc		
Know that zinc should be given to child for		
Acute diarrhoea	37.8	51.0
Persistent diarrhoea	79.8	70.1
Administration of zinc		
Know that zinc should be given to a child withdiarrhoea for at least 10 days	53.3	77.3
Belief about effectiveness of zinc treatment		
Zinc tablets are effective for treatment of diarrhoea	68.8	80.5
Zinc reduces duration of diarrhoea episode	76.3	92.4
Zinc helps strengthen the immune system of child	69.0	89.0
Zinc helps reduce severity of diarrhoea	65.8	83.7
Zinc reduces risk of new diarrhoea episode in the following 2-3 months	58.4	85.2

*The total number of caregivers whose children received zinc treatment for diarrhoea in the last two weeks. It is smaller than the number of children who received zinc treatment because some caregivers cared for more than one child

The survey respondents had generally a good perception of the effectiveness of zinc treatment for diarrhoea. Table 3 shows that more than two-thirds of the caregivers who had ever heard about zinc believed that zinc tablets were effective in treating diarrhoea. Moreover, 76% knew that zinc reduces the duration of a diarrhoea episode; 69% reported that zinc helps strengthen the child's immune system; and 66% mentioned that zinc helps reduce the severity of diarrhoea. The reaction was even more positive among those who used zinc: more than 80% of the users of zinc mentioned that zinc was effective, and most knew that zinc reduces the duration of diarrhoea (92%), severity (84%), and the risk of subsequent new diarrhoea episodes (85%).

### Association between recall of communication message and zinc-related knowledge andbehaviour

Over two-thirds (68%) of the respondents had heard at least one message relating to prevention and treatment of diarrhoea in the past three months, primarily from radio (52%) and television (68%). Only 13% heard messages relating to treatment of diarrhoea from a community health worker. Over half (53%) had heard or seen a message specifically about zinc, primarily on television (85%) or radio (50%). Recall of messages was significantly associated with the frequency of exposure to radio or to television (data not shown). Residents of the three initial POUZN pilot districts (Kathmandu, Lalitpur, and Bhaktapur) were exposed to radio messages about use of zinc for approximately one year before the mass-media campaign of the project extended to television and to the additional 27 districts nationwide. Residents in these three districts were significantly more likely to recall any zinc-related messages from television or radio than were residents of the remaining 27 districts (63% vs 46% respectively, p<0.0001).

**Fig. 3.**
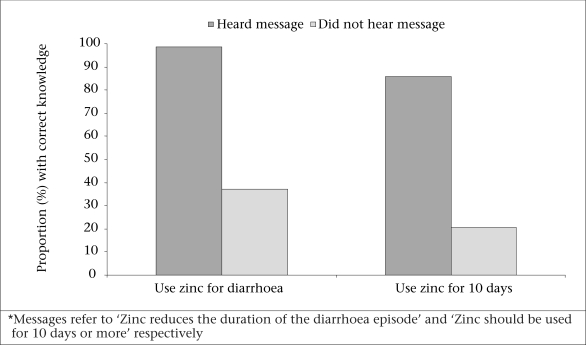
Zinc-related knowledge by recall of relevant message*

There was a positive association between recall of relevant message and zinc-related knowledge. Most (98.5%) respondents who reported that they had heard the message—‘Zinc reduces the duration of the diarrhoea episode’—via radio or television knew that they should use zinc for acute or persistent diarrhoea whereas only 37% of the respondents who had not heard the same message knew that they should use zinc to treat diarrhoea (Fig. 3). Similarly, 86% of the respondents who had heard the message—‘Zinc should be used for 10 days’—knew that zinc should be used for 10 days compared to 20% of those who had not heard this specific message.

**Fig. 4.**
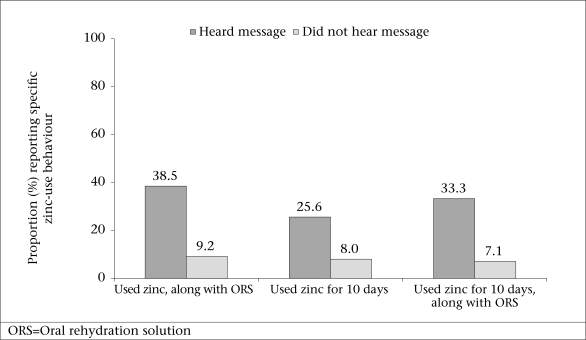
Zinc-use behaviour by recalling the message of correct use

Exposure to relevant message was also strongly associated with correct zinc-use behaviours. The respondents who could recall the correct use of a specific message were significantly more likely to use zinc correctly than those who could not (Fig. 4). The respondents who heard the message of correct use were almost five times more likely to use zinc correctly (i.e. with ORS and for 10 days) than respondents who had not heard the message.

### Predictors of use of zinc

Multiple logistic regressions were used for examining the variables associated with two dependent variables: (a) use of zinc and (b) correct use of zinc (i.e. with ORS and for at least 10 days) for the treatment of paediatric diarrhoea in this population. The sample for these analyses was limited to children (n=289) who had diarrhoea in the two weeks preceding the survey. Table 4 presents the results of multiple logistic regression analysis on use of zinc and correct use of zinc for predictors that were statistically significant. Children in the highest wealth quintile were more likely to be treated with zinc than children in the lowest wealth quintile [odds ratio (OR)=5.76, 95% confidence interval (CI) 5.06-6.55]. Children whose caregivers had secondary education or higher were also more likely to be treated with zinc (OR=1.76, 95% CI 1.27-2.44). Residents in the three project pilot districts were significantly more likely to use zinc (OR=2.07, 95% CI 1.32-3.24). The likelihood of using zinc increased significantly if the caregivers were exposed to any zinc-related message, had positive attitudes towards effectiveness of zinc treatment, or believed that zinc could be easily obtained.

**Table 4.** Percentage distribution of selected characteristics of caregivers of children, aged less than six years, with diarrhoea (who used zinc and who used zinc correctly) and odds ratios from multivariate logistic regression models examining association of selected characteristics of caregivers of children, aged less than six years, with diarrhoea (n=289) with use of zinc and correct use of zinc, Nepal, 2008

Independent variable	Use zinc		Use zinc correctly			Total no. of respondents
	%	OR (95% CI)	%	OR (95% CI)		
Characteristics of caregivers						
Age of caregiver (mean)	33	0.99 (0.90-1.10)	41	0.91 (0.83-0.98)		289
Education of caregiver						
No education (ref)	4.9	1.00	1.9	1.00		129
Primary education	9.0	1.09 (0.87-1.38)	2.1	1.53 (0.22-10.77)		55
Secondary education	31.6	2.73 (0.95-7.88)	19.1	5.31 (0.41-68.15)		99
Higher than secondary education	33.6	1.76 (1.27-2.44)	21.6	2.46 (0.64-9.41)		6
Household wealth quintile						
Poorest (ref)	2.4	1.00	1.8	1.00		61
Middle-poor	6.6	2.33 (1.34-4.05)	4.2	1.39 (0.19-10.37)		60
Middle	8.2	1.73 (0.31-9.69)	1.6	0.39 (0.05-3.32)		58
Middle-rich	27.3	5.32 (0.99-28.56)	15.9	1.54 (0.13-18.14)		70
Richest	38.0	5.76 (5.06-6.55)	20.5	1.59 (0.35-7.25)		40
Being in three pilot districts*						
In remaining 24 districts (ref)	4.8	1.00	3.0	1.00		220
In three pilot districts	10.6	2.07 (1.32-3.24)	0.1	0.65 (0.19-2.20)		63
Caregiver's exposure tomass-media message†						
Not exposed to relevant message about zinc (ref)	2.9	1.00	7.1	1.00		136
Exposed to relevant message about zinc	26.5	2.13 (1.81-2.51)	33.3	2.02 (1.85-2.19)		153
Caregiver's knowledge andbeliefs about zinc‡						
Disagree that zinc is an effective treatment for diarrhoea (ref)	5.9	1.00			176	
Agree that zinc is an effective treatment for diarrhoea	30.2	1.92 (1.07-3.46)			113	
Perceive that zinc is not easy to obtain (ref)	7.6	1.00	1.5	1.00	194	
Perceive that zinc is easy to obtain	31.3	2.10 (1.22-3.61)	16.8	1.76 (1.49-2.09)	95	

*Three POUZN pilot districts include Kathmandu, Lalitpur, and Bhaktapur. Caregivers in these three districts were exposed for a longer time to zinc-related messages via a radio campaign;

†Zinc-use message refers to any zinc-related message recalled; correct message of use of zinc refers to recalling both messages ‘Zinc should be used with ORS’ and ‘Zinc should be used for at least 10 days’;

‡This variable was not significant in the regression for correct use of zinc; so, it is excluded from the final model.

CI=Confidence interval;

OR=Odds ratio;

ref=Reference group

The logistic regression analysis detected only a few significant predictors of correct use of zinc. Children who had a younger caregiver (OR=0.91, 95% CI 0.83-0.98), whose caregiver was exposed to the mass-media message that zinc should be used for 10 days (OR=2.02, 95% CI 1.85-2.19), or whose caregiver perceived that zinc is easy to obtain (OR=1.76, 95% CI 1.49-2.09) were more likely to be treated with zinc for the recommended 10 days, along with ORS as recommended.

## DISCUSSION

This survey is one of the few published to examine current paediatric diarrhoea-treatment practices, with a focus on knowledge and use of zinc, in areas with large-scale zinc-promotion programmes. Although the findings of this household survey are specific to the context in Nepal, many merit consideration in planning zinc-promotion programmes elsewhere.

It should be noted that 15% of the caregivers in this population had used zinc during their child(ren)'s most recent episode of diarrhoea. While the overall use of zinc was relatively low, the 2006 Nepal Demographic and Health Survey (DHS) had shown that use of zinc nationwide for paediatric diarrhoea was just 0.4% (13). Due to its sampling design, the baseline DHS data cannot be analyzed for just the 30 districts included in this survey but given that zinc was not available or promoted in the study districts until 2006, it may be assumed that there was virtually no use of zinc in the survey districts until the MoHP, UNICEF, NFHP, and POUZN project began their combined efforts during 2007-2008. While there is still room for significant improvement, initial results were promising and suggest that the combined public/private-sector approach to promoting use of zinc could be successfully implemented in similar settings with a high prevalence of diarrhoea.

It was encouraging to find that 68% of the caregivers used ORS to treat paediatric diarrhoea. This finding may reflect the fact that ORS has been promoted heavily in Nepal for many years and is consistent with data from Bangladesh (10). Our survey indicated that ORS/ORT was still the most popular treatment used for paediatric diarrhoea, and most users of zinc also used ORS/ORT as recommended. The use of ORS/ORT in our survey was higher than the level of its use reported in the 2006 DHS (13). Due to the cross-sectional nature of this survey, it was not possible to quantify the contributions that the efforts of the MoHP and other health-related projects, including POUZN, to promote use of ORS/ORT might have had during this time period. Zinc-intervention programmes should be carefully designed to promote the use of zinc, along with ORS/ORT, and ultimately improve all diarrhoea-management practices.

Many caregivers have not yet absorbed the message that ORS should be used in conjunction with zinc, which was reflected by the result that only half of those who heard about zinc messages recalled that zinc and ORS should be used together. This may be due, in part, to certain segments of the population not hearing zinc-promotion messages and, thus, remaining entirely unaware of the importance of use of zinc. This suggests that additional communication channels may be necessary to reach popu-lations that are not as easily reached by mainstream media.

While gaps in knowledge and use of zinc remain, our survey did find a positive association between exposure to zinc-related campaign-messages and improved knowledge and practice of use of zinc. The likelihood of using zinc—both at all and correctly—was significantly increased if a caregiver was exposed to and subsequently recalled the mass-media messages on zinc. Most users of zinc either correctly used zinc with ORS or correctly used zinc for 10 days; however, only half of all the caregivers correctly administered zinc with ORS for the full 10 days as recommended. This highlights the importance of ensuring that communication messages focus on the basic but key information about correct use when introducing a new product.

Additionally, other important information should be conveyed through the communication channels, encouraging caregivers’ compliance with the 10-day protocol or reinforcing information on inappropriate antibiotic or antidiarrhoeal drug-use. The results of the survey indicate that a high proportion of children were treated inappropriately with antibiotics, antidiarrhoeals, and other non-specified pills and syrups. These findings were consistent with national-level data from the 2006 DHS, which showed that other and unknown pills and syrups were used for treating 36% of paediatric diarrhoea cases (13). Antibiotic therapy is not the most effective treatment for diarrhoea unless there is apparent blood in stool and antidiarrhoeals are ineffective and, in some cases, dangerous for under-five children.

### Limitations

Some limitations of the study should be considered when interpreting the findings. First, without baseline data available for the study districts, it is not possible to directly attribute apparent increases in use of zinc to the MoHP/UNICEF/NFHP/POUZN efforts. Baseline data were not available for knowledge or recollection of zinc or other diarrhoea-treatment messages; so, we could not evaluate any trends in these parameters, and no repeat surveys were conducted. Future programmatic efforts should incorporate data-collection activities to help determine whether additional or different approaches lead to increased knowledge and use of zinc. The small number of children reported to have diarrhoea in the last two weeks is another limitation: the small sample-size could influence the power of the study, limiting the ability to detect some significant variables. All data were based on self-reported information, and, thus, the potential for recall bias exists. Finally, due to the cross-sectional design, it is not possible to infer a causal relationship between specific variables and use of zinc.

### Conclusions

The experience of the POUZN project in Nepal has shown that the private sector has an important role to play in promoting zinc treatment and distributing products. Mass media were shown to be very effective in increasing knowledge of child's caregiver about zinc and its use. Given the media's power to influence health-related behaviours, programmes should develop and disseminate messages that encourage both caregivers’ and care providers’ compliance to the recommended zinc protocols. However, our findings also demonstrated that focused efforts must be made in future programmes to address factors that motivate care providers to recommend or provide antibiotics and antidiarrhoeals inappropriately. With continual evidence-based refinement of project approaches and communication strategies, the potential exists to reach even more caregivers and increase the use of zinc with ORS/ORT, leading to reduced morbidity and mortality in communities with high levels of paediatric diarrhoea.

## ACKNOWLEDGEMENTS

The study was supported by the United States Agency for International Development (USAID) (Contract No. GPO-I-00-04-00007, Task Order 5). The authors acknowledge Dr. Y.V. Pradhan for his vision and support to the zinc programme in Nepal and USAID/Nepal and USAID/Washington for their leadership and financial support. The authors also acknowledge the contributions of Susan Mitchell and Slavea Chankova of Abt Associates Inc. for their review of and contributions to the research report. In addition, the authors thank the Population Services International team in Nepal, led by Steven Honeyman. The authors also thank Blitz Media of Kathmandu for collecting data used in this analysis.

## References

[1] United Nations Children's Fund (2009). Diarrhoea: why children are still dying and what can be done.

[2] Bhutta ZA, Black RE, Brown KH, Gardner JM, Gore S, Hidayat A (1999). Prevention of diarrhea and pneumonia by zinc supplementation in children in developing countries: pooled analysis of randomized controlled trials. Zinc Investigators’ Collaborative Group. J Pediatr.

[3] Bhutta ZA, Bird SM, Black RE, Brown KH, Gardner JM, Hidayat A (2000). Therapeutic effects of oral zinc in acute and persistent diarrhea in children in developing countries: pooled analysis of randomized controlled trials. Am J Clin Nutr.

[4] Fontaine O (2001). Effect of zinc supplementation on clinical course of acute diarrhoea. J Health Popul Nutr.

[5] Baqui AH, Black RE, El Arifeen S, Yunus M, Chakr-aborty J, Ahmed S (2002). Effect of zinc supplementation started during diarrhoea on morbidity and mortality in Bangladeshi children: community randomised trial. BMJ.

[6] World Health Organization (2004). Clinical management of acute diarrhoea: WHO/UNICEF Joint Statement.

[7] Ellis AA, Winch PJ, Daou Z, Gilroy KE, Swedberg E (2007). Home management of childhood diarrhoea in southern Mali—implications for the introduction of zinc treatment. Soc Sci Med.

[8] Winch PJ, Gilroy KE, Doumbia S, Patterson AE, Daou Z, Coulibaly S (2006). Prescription and administration of a 14-day regimen of zinc treatment for childhood diarrhea in Mali. Am J Trop Med Hyg.

[9] Winch PJ, Doumbia S, Kante M, Male AD, Swedberg E, Gilroy KE (2008). Differential community response to introduction of zinc for childhood diarrhea and combination therapy for malaria in southern Mali. J Nutr.

[10] Larson CP, Saha UR, Nazrul H (2009). Impact monitoring of the national scale up of zinc treatment for childhood diarrhea in Bangladesh: repeat ecological surveys. PLoS Med.

[11] Armand, F (2003). Social marketing models for product-based reproductive health programs: a comparative analysis.

[12] Janz NK, Champion VL, Stecher VJ, Glanz K, Rimer BK, Lewis FM (2002). Health behavior and health education: theory, research and practice. The health belief model, 3rd ed..

[13] Nepal (2007). Ministry of Health and Population. Nepal demographic and health survey 2006.

[14] Filmer D, Pritchett L (2001). Estimating wealth effects without expenditure data—or tears: an application to educational enrollments in states of India. Demography.

[15] Rutstein SO, Johnson K (2004). The DHS wealth index.

[16] Bouis H (1994). The effect of income on demand for food in poor countries: Are our food consumption databases giving us reliable estimates?. J Dev Econ.

